# Culturally Safe eHealth Interventions With Aboriginal and Torres Strait Islander People: Protocol for a Best Practice Framework

**DOI:** 10.2196/34904

**Published:** 2022-06-10

**Authors:** Georgina R Chelberg, Kaley Butten, Ray Mahoney, eHRCATSIH Group

**Affiliations:** 1 Australian E-Health Research Centre Commonwealth Scientific and Industrial Research Organisation Herston Australia; 2 Centre for Online Health The University of Queensland Woolloongabba Australia; 3 School of Public Health The University of Queensland Herston Australia; 4 See Acknowledgments

**Keywords:** eHealth, mHealth, telehealth, Aboriginal, Indigenous, First Nations, best practice, Australia, development, framework, Torres Strait Islander, co-design, culturally safe, culturally sensitive, evaluation, health care model

## Abstract

**Background:**

There is growing global evidence on the adoption and effectiveness of eHealth (including mobile health and telehealth) by First Nation peoples including Aboriginal and Torres Strait Islander people. Although there are frameworks to guide eHealth development, implementation, and evaluation, it is unknown whether they adequately encapsulate the health, cultural, and community-related priorities of Aboriginal and Torres Strait Islander people.

**Objective:**

The aim of this research program is to prepare a best practice framework that will guide the co-design, implementation, and evaluation of culturally safe eHealth interventions within existing models of health care for Aboriginal and Torres Strait Islander people. The framework will be a synthesis of evidence that represents best practices in eHealth, as determined by Aboriginal and Torres Strait Islander people.

**Methods:**

Research activities to develop the best practice framework will occur in stepped but overlapping qualitative research phases with governance from an existing multiagency research collaboration (the Collaboration). The research protocol has been informed by key research frameworks such as the SPIRIT (Standard Protocol Items: Recommendations for Interventional Trials) and Developers of Health Research Reporting Guidelines. The seven phases of research will include the following: systematic literature review, scoping review, theme development, theme consultation, Delphi processes for expert reviews, and dissemination.

**Results:**

Members of the Collaboration conceived this research program in August 2020, and a draft was produced in June 2021 with subsequent funding obtained in July 2021. The Collaboration approved the protocol in December 2021. Results for several research phases of the best practice framework development are expected by January 2023, commencing with the systematic literature review and the scoping review.

**Conclusions:**

The research program outlined in this protocol is a timely response to the growing number of eHealth interventions with Aboriginal and Torres Strait Islander people. A best practice framework is needed to guide the rigorous development and evaluation of eHealth innovations to promote genuine co-design and ensure cultural safety and clinical effectiveness for Aboriginal and Torres Strait Islander people.

**International Registered Report Identifier (IRRID):**

DERR1-10.2196/34904

## Introduction

### eHealth Interventions

eHealth broadly refers to the delivery and management of health care using a range of information and communication technologies that connect consumers with health professionals (see Multimedia Appendix 1) [1-3]. The term eHealth is used throughout this paper to encompass modalities that involve interaction between health professionals and consumers, including telehealth, mobile health (mHealth), videoconferencing, smart technology platforms, and remote monitoring.

eHealth’s strength lies in its ability to connect consumers and health care professionals, who are often separated geographically. However, geographical separation is not the sole driver for the adoption of eHealth. Since early 2020, the global impact of COVID-19 has fast-tracked innovation in health service delivery and highlighted eHealth as a critical resource not only to reduce exposure to and the spread of infectious disease but to enable continuity of health care more broadly [4-6]. Many consumers and caregivers experience the value of eHealth when social, cognitive, or mental health conditions pose additional challenges to accessing health services. eHealth is enabling mental health care [5,7] and support for a range of chronic illnesses such as diabetes and heart disease within clinical [8-10] and in-home settings [11,12]. Comparable or superior clinical effectiveness of eHealth has been established for some modalities such as telehealth [8,13], with emerging evidence for others including mHealth platforms and text messaging [9,14,15]. Furthermore, digital health innovations may facilitate health access for culturally diverse populations despite systemic barriers including narrow conceptualizations of health, English as the dominant language, racism, and discrimination [10,16,17].

### eHealth with Aboriginal and Torres Strait Islander People

There is growing global evidence on the adoption of digital technologies by First Nation peoples [10,18-20]. Australian research has shown eHealth can address access challenges associated with health care for Aboriginal and Torres Strait Islander people. For example, telehealth services can help avoid the distress of separation from Country and kin by reducing the need for in-person travel to primary or tertiary care appointments that may involve navigation of unfamiliar environments [18,21]. Additionally, eHealth may reduce costs associated with patient travel, operating costs for the service provider [22], and enable the expansion of services offered by Aboriginal and Torres Strait Islander primary care and community organizations [18,23]. Research also notes that eHealth interventions can increase family involvement in health care [24] and that family inclusion can enhance engagement with eHealth [3]. Effective and engaging mental health interventions such as *AIMhi Stay Strong*, and *iBobbly* demonstrate the relevance of eHealth and significance of co-design processes with Aboriginal and Torres Strait Islander people [20]. Both these mobile apps have undergone further research and development following the positive outcomes of pilot testing [25-27]. A recent app development and feasibility trial regarding social and emotional well-being with Aboriginal and Torres Strait Islander women reported mixed results for adoption, engagement, and user feedback [28].

### Implementation and Evaluation of eHealth

eHealth frameworks guide the development of platforms and lift the rigor of implementation, evaluation, and reporting. Foundational works by Eng et al [29] on the “Evaluation of Interactive Health Communication” have been complemented by other key guides including the CONSORT (Consolidated Standards of Reporting Trials) statement [30], the Mobile health evidence and Reporting Assessment (MeRA) [31], Mobile Application Rating System [32], the Centre for eHealth Research and Development roadmap [33], Model for Assessment of Telemedicine [34], and ongoing work in digital health implementation by Greenhalgh et al [35,36]. Application of these frameworks increases the potential impact and relevance of eHealth, including its contribution to addressing health inequalities [33,37].

### Culturally Safe eHealth With Aboriginal and Torres Strait Islander People

The frameworks, however, may not accurately reflect the values and priorities of the culturally diverse populations for which they may be intended. For example, Aboriginal and Torres Strait Islander people conceptualize health and well-being as dynamic, holistic, and interconnected, in contrast to the dominant biomedical approach [38-40] on which the majority of health interventions are based. Extensive eHealth research conducted by Maar et al [41] with First Nations communities of Canada has emphasized the need for respect and commitment to community priorities, worldviews, and culture throughout the research process. Therefore, it is critical to acknowledge that western-derived frameworks are not directly transferrable to Aboriginal and Torres Strait Islander health settings and such research must be embedded with culturally respectful approaches [41-43].

A culturally safe framework for eHealth evaluation is also significant because of the persistent health inequities between Aboriginal and Torres Strait Islander people and non-Indigenous people. Complex interactions between racism, marginalization, and rapid social and economic change continue to perpetuate gross inequities in health service provision, health outcomes, as well as health and well-being for Aboriginal and Torres Strait Islander people in Australia [44-46]. Furthermore, although eHealth adoption and evidence of its effectiveness are increasing, there remains a global “digital divide” (or “digital poverty”), where persons who may benefit most from eHealth face persistent and complex barriers to access these services [47]. These barriers include socioeconomic challenges, increasing age, chronic illness, education including health literacy, ethnicity, remoteness, and digital literacy [3,19,48-51]. Such factors are crucial considerations in the co-design, trial, implementation, and evaluation of eHealth interventions.

Therefore, it is unknown whether existing eHealth implementation or evaluation frameworks adequately encapsulate the health, cultural, and community-related priorities of Aboriginal and Torres Strait Islander people.

Although scientific reviews [10,19,20] and recommendations for application of eHealth in diverse cultures have been published [3,41,50], there is currently no comprehensive research, implementation, and evaluation guide. Consequently, eHealth interventions with Aboriginal and Torres Strait Islander people have routinely been brief, single studies that lack authentic alignment with co-design principles [52] and yield low-grade evidence for health outcomes. The World Health Organization observes this as a weakness of the broader global eHealth movement, which has “…driven a proliferation of short-lived implementations and an overwhelming diversity of digital tools, with a limited understanding of their impact on health systems and people’s well-being” [37]. Consensus from public health research has also noted the critical lack of quality standards and evidence-based content for most health-based apps, particularly those stating clinical benefits [53-55]. Sustainable and relevant eHealth interventions are needed where genuine co-design incorporates multidisciplinary expertise, end-user perspectives, and addresses the health priorities of Aboriginal and Torres Strait Islander communities in line with foundational research principles [42,43].

The overall aim of this research program is to prepare a best practice framework that will guide the co-design, implementation, and evaluation of culturally safe eHealth interventions within existing models of health care for Aboriginal and Torres Strait Islander people. A best practice framework can be described as a synthesis of key elements that will ensure best practices in real-world settings, beyond empirical studies [56]. Therefore, the proposed framework will be a synthesis of evidence that represents the best practices in eHealth, as determined by Aboriginal and Torres Strait Islander people.

## Methods

### Research Partners

It is imperative that holistic and cultural values of health and well-being are upheld in eHealth research with Aboriginal and Torres Strait Islander people. The Collaboration acknowledges the expertise and leadership of Aboriginal and Torres Strait Islander Community Controlled Health Organisations (ATSICCHOs) in the delivery of primary care across Australia [57,58]. ATSICCHO models of care with Aboriginal and Torres Strait Islander people are centered on connections to culture, Country, and kin [59-61]. Research partnerships with ATSICCHOs and other community-controlled organizations in Queensland and the Northern Territory have been developed through ongoing relationships and consultations by the Collaboration members. Participants from the partnering organizations for the research activities outlined in this protocol will include stakeholders, hereby used to refer to doctors, Aboriginal and Torres Strait Islander health workers, nurses, administration staff, research personnel, board members, clients, and other community members.

### Ethical Considerations

Certain phases of the research outlined in this protocol, such as the recruitment of experts for the Delphi processes, will require ethical approval from a Human Research Ethics Committee (HREC). As the Collaboration has ongoing partnerships with ATSICCHOs and other community-controlled organizations, other phases involving feasibility studies will have their own HREC approval and research protocol.

### Theoretical Frameworks and Design

The scientific research questions (RQs) guiding this program of research are as follows:

RQ1: What is the scientific evidence for determining what is important to Aboriginal and Torres Strait Islander people in the adoption, engagement, and evaluation of eHealth interventions within an Aboriginal and Torres Strait Islander health context?

RQ2: What existing frameworks and practice guidelines should be used to inform eHealth interventions with Aboriginal and Torres Strait Islander people? (ie, drawing on Aboriginal and Torres Strait Islander knowledge and accepted guidelines)

RQ3: What principles and values are important to Aboriginal and Torres Strait Islander stakeholders (consumers, facilitators, etc) when co-designing and using eHealth?

RQ4: How can the above outcomes be integrated to inform a set of principles for best practices (facilitation and reporting) in eHealth interventions within the Aboriginal and Torres Strait Islander health context?

**Figure 1:**
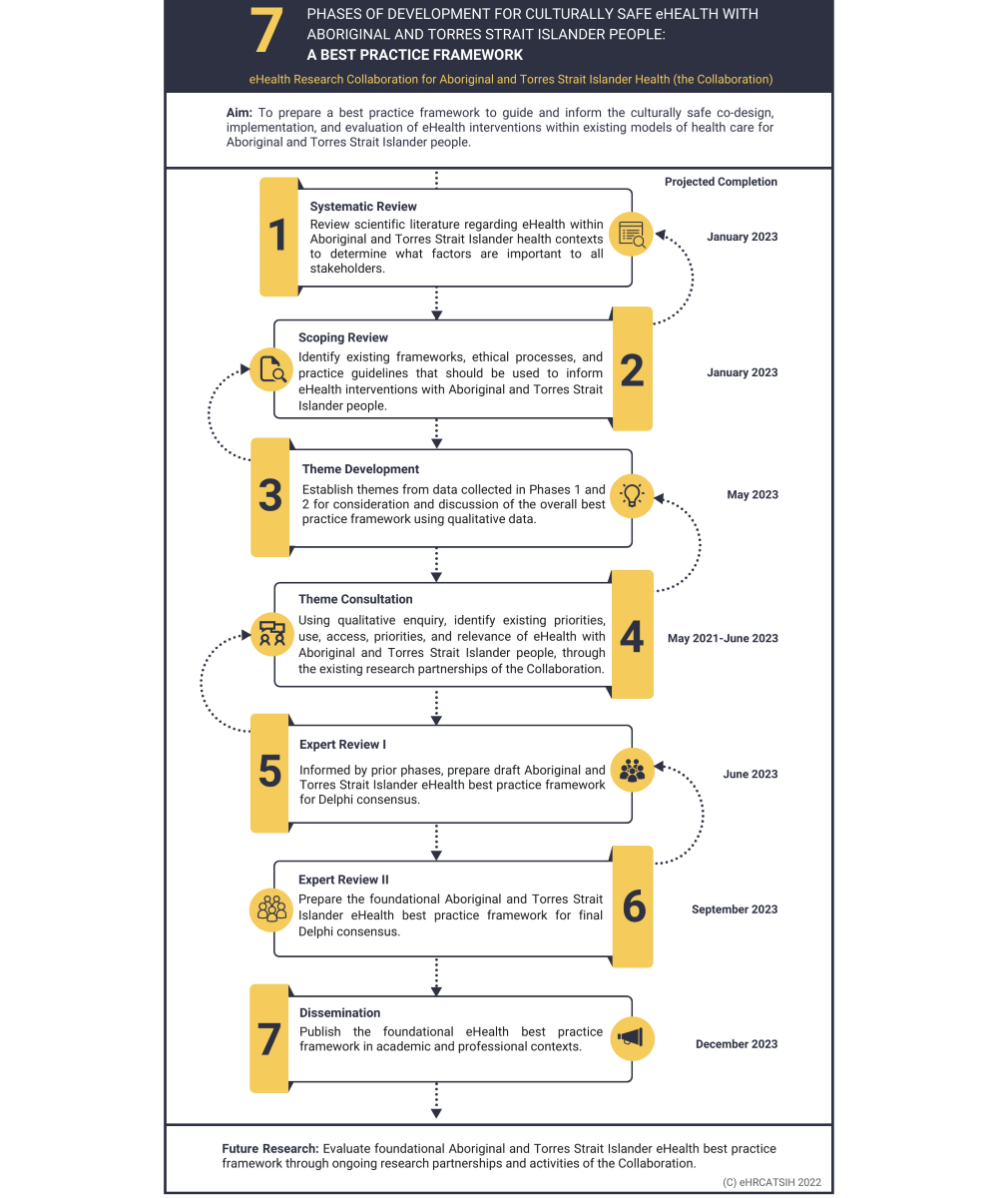
Developmental phases of the eHealth best practice framework.

The structure of this protocol was informed by SPIRIT (Standard Protocol Items: Recommendations for Interventional Trials) [62]. Although intended for clinical trials, this checklist provides research transparency and ensures that key research elements are addressed. Data collection processes and development of the best practice framework will occur in stepped but overlapping qualitative research phases (see Figure 1) informed by the work of Moher et al [63]. Each research phase draws on additional frameworks such as PRISMA (Preferred Reporting Items for Systematic reviews and Meta-Analyses) [64], Delphi approaches [65-67], and scoping frameworks [68-70]. Additional reference has been made to relevant scientific publications regarding the development of practice guidelines [71-76] and health research protocols [74,77,78] throughout the development of this protocol. Further details of each of the 7 research phases are outlined as follows.

### Phase 1: Systematic Literature Review

#### Study Aim

The aim of this systematic literature review will be to identify the characteristics of eHealth facilitation, implementation, and adoption with the goal of determining what factors are important to Aboriginal and Torres Strait Islander people and determine gaps in the literature. This study will address RQ1. A full protocol for this systematic review will be registered with the International Prospective Register of Systematic Reviews and follow the PRISMA guidelines for reporting a systematic literature review [64].

#### Searches, and Inclusion and Exclusion Criteria

Recommendations by the Lowitja Institute [79] and university librarians will refine the search terms and strings to capture all iterations of eHealth interventions with Aboriginal and Torres Strait Islander people. eHealth search terms will be based on keywords including eHealth, telehealth, telemedicine, remote monitoring, mHealth, Internet of Things, and smart technology. Electronic database searches will be conducted on Cochrane, Embase, CINAHL, PubMed, Scopus, Web of Science, and PsycINFO, and they will be limited to full-text papers in English, with no limit regarding the publication date. Participants of the intervention studies of all ages will be either Aboriginal and Torres Strait Islander people or health staff (either Aboriginal and Torres Strait Islander or non-Indigenous) who work with Aboriginal and Torres Strait Islander people; if the participants are culturally diverse, the outcomes specific to Aboriginal and Torres Strait Islander people are reported. As per the aim of this systematic review and the scope of the overall research protocol, studies that relate to eHealth interventions with other First Nations populations will be excluded. Studies will be focused on an eHealth intervention (as defined in the Introduction) and report on one or more of the following outcomes: adoption, implementation, integration, use (usage data), user perspectives (eg, feedback, knowledge, level of support, barriers, enablers, accessibility, and acceptability). Experimental, quasi-experimental, and qualitative studies from peer-reviewed scientific journals will be included. However, due to the potentially high yield of search results, separation of the experimental/quasi-experimental from qualitative studies may occur, with 2 systematic literature reviews produced. Database searches, screening, and data extraction will be conducted by 2 researchers.

#### Quality Assessment

Risk of bias and methodological quality for the experimental and quasi-experimental studies will be evaluated by at least 2 authors using the Joanna Briggs Institute (JBI) Critical Appraisal Checklist [80] and Level of Evidence tool [81].

#### Analysis and Reporting

A descriptive approach will be used for data synthesis due to the mix of study designs and research approaches expected across eHealth interventions. Descriptive analysis will consider the intervention(s) (modality, exposure, etc), health challenge(s) addressed, clinical outcomes, use outcomes, user-feedback outcomes, and characteristics of the intervention process that are important to Aboriginal and Torres Strait Islander people. These embedded factors will be identified through thematic analysis. For example, stakeholders within an eHealth intervention may highlight “access to technology” or “co-design” as significant factors in the application of digital health with Aboriginal and Torres Strait Islander people.

Findings of the systematic literature review(s) will inform subsequent phases of the research program for development of the best practice framework. Outcomes will also be disseminated in a scientific journal paper and at a relevant conference. Phase 1 is projected to be completed by January 2023.

### Phase 2: Scoping Review

#### Aim and Rationale

The aim of the scoping review is to identify what existing frameworks, ethical processes, and practice guidelines, particularly grounded with Aboriginal and Torres Strait Islander peoples’ knowledge, should be used to inform eHealth interventions with Aboriginal and Torres Strait Islander people, and to identify gaps in the scientific and gray literature. For example, a known gap in the literature is that the MeRA checklist has not been validated with Aboriginal and Torres Strait Islander populations. A central component of this review is to identify resources, solutions, and processes enriched with the voices, priorities, and wisdom of Aboriginal and Torres Strait Islander people. Therefore, the review seeks to identify the available frameworks, guidelines, and practice principles across the eHealth field, as well as those guiding health implementation and evaluation with Aboriginal and Torres Strait Islander people more broadly. Examples of these include the national ethical guidelines [42,82,83] and bespoke reference documents such as the South Australian Aboriginal Health Research Accord [84]. Although a scoping process was conducted earlier by the authors, this full scoping review will ensure that all literature has been captured systematically. This study will address RQ2.

#### Scoping Design

Broadly speaking, the research team envisages the scoping process to follow standard practices, as outlined in the updated JBI iterations [70,85] of the original work by Arksey and O’Malley and Levac et al [68,69]. The scoping review research stages will achieve in-depth and broad scoping results that will identify all relevant literature, regardless of study design. Importantly, this will include a parallel process of iterative consultation with experts to source additional practice insights. A full protocol for the scoping review will be date-stamped on the Open Science Framework.

#### Searches, and Inclusion and Exclusion Criteria

Electronic searches will be conducted on the Cochrane, Embase, CINAHL, PubMed, Scopus, Web of Science, and PsycINFO databases. Search terms and strings will be finalized using recommendations by the Lowitja Institute and university librarians to capture iterations of health interventions involving Aboriginal and Torres Strait Islander people. The following keywords will form the basis of the search: framework, practice guide, best practice, principles, and recommendations. Experimental, quasi-experimental, and qualitative papers will be sought in English, with no limit on the date of publication. Gray literature will be researched to identify existing resources in health care, health organizations, and health entities, via health, nongovernment, and government websites.

Searches and screening will be conducted by 2 researchers with discussion for resolving differences. Manual searches of reference lists (both scientific and gray literature) will be conducted to identify unique results or resources. Websites and other internet-based resources will be identified and screened using a tabulated spreadsheet. A final list of full-text papers and their citations that meet the inclusion criteria will be downloaded and saved.

#### Data Extraction and Synthesis

Relevant data from the retained papers and resources will be tabulated in a spreadsheet. The data items of relevance will include Aboriginal and Torres Strait Islander collaborative groups and entities, the region of Australia, health focus, characteristics of interventions, frameworks/guidelines/best practices, rationale/feedback/discussion regarding the application of frameworks, and participant feedback specific to the frameworks/research approaches.

A descriptive approach will be used for data synthesis due to the expected mix of study designs and gray literature results. Descriptive analysis will consider the identified frameworks, best practices, and guidelines, as well as their application to the setting and the health intervention (including eHealth). Thematic analysis will highlight the themes and inclusions that researchers and participants nominate or reflect as critical to culturally safe health interventions. The PRISMA-ScR (Preferred Reporting Items for Systematic reviews and Meta-Analyses extension for Scoping Reviews) will be used for quality reporting of the scoping study.

#### Dissemination

Findings of the scoping review will inform subsequent phases of the research program for development of the best practice framework. Outcomes will also be disseminated in a scientific journal paper and at a relevant conference. The projected completion of Phase 2 is January 2023.

### Phase 3: Theme Development

Informed by the findings of Phases 1 and 2, broad themes for consideration will be drafted and used to prompt and support deliberation and discussion of the overall best practice framework using qualitative data collection methods. Appraisal of the data from Phases 1 and 2 will be conducted by members of the Collaboration with HREC approval if warranted. Members of the Collaboration are mentioned in the Acknowledgements. Criteria for themes will be established in Phases 1 and 2 using the empirical evidence available, as well as the professional judgement and considering the context of the relevant research activities underway within the Collaboration. Phase 3 is expected to be completed in May 2023.

### Phase 4: Theme Consultation

#### Study Aim and Rationale

The aim of this qualitative research is to identify the perceptions and experiences of stakeholders about the use, access priorities, and relevance of eHealth with Aboriginal and Torres Strait Islander people and to generate overall themes. The qualitative research activities will be embedded within existing eHealth research studies by members of the Collaboration. This pragmatic data will complement the themes derived from the literature in Phases 1 to 3.

#### Study Design and Data Collection

Semistructured interviews and focus groups will be used to explore the perceptions and experiences of stakeholders within the research partnerships and activities of the Collaboration. Specific inclusion/exclusion criteria and recruitment strategies will be outlined in each of the eHealth study protocols with HREC approval and published in due course. For example, a mixed method feasibility trial is currently being conducted for an mHealth platform to manage hypertension and other cardiovascular risk factors with stakeholders from 2 ATSICCHOs in Queensland (Mahoney and Goodman, 2021; HREC/2021/QCH/61500). Qualitative enquiry conducted within the mHealth platform feasibility study includes a core set of questions about eHealth that will be replicated in other feasibility studies led by the Collaboration. The overall line of enquiry for the qualitative activities will be published in due course. Although themes from Phase 3 will be available to support the qualitative process, the methods for data collection and analysis will be phenomenologically informed, whereby the phenomena of eHealth and user needs are explored, described, and interpreted as they relate to the individuals and their own experiences. A phenomenological approach is regularly used in qualitative health research to understand health care user and service provider experiences because it prioritizes the voices and narratives of the participants as opposed to the interviewer and line of questioning [86]. This approach is particularly important as we aim to understand if there are culturally specific needs and values related to eHealth implementation and adoption.

#### Analysis and Synthesis of Findings

Interviews and focus groups will be audio-recorded and transcribed in preparation for thematic analysis. Although there are synergies across the Collaboration’s research activities and feasibility trials, study investigators will conduct their respective data collection and analyses independently. Resulting themes and research outcomes will be cross-referenced across studies in cooperation with other study investigators, only after the conclusion of the data collection and analysis to prevent crossover of research findings. Scientific dissemination of the broader research design, study participants, interventions, outcomes, and conclusions for each eHealth feasibility study will be coordinated by study investigators. Specifics relevant to the reporting of the resulting best practice framework will be documented accordingly. Phase 4 is projected to be completed in June 2023.

### Phase 5: Expert Review I

The concepts and overall themes defined up to this point (Phases 1 to 4) will be put forth in a draft document with a ranking scale as part of a Delphi exercise. The Delphi consensus method is a systematic process where items can be refined based on expert opinion and consensus [65,67]. Members of the Collaboration will participate in the first review of the concepts that will form the first draft of the best practice framework. In subsequent Delphi rounds, members of the Collaboration will recruit subject matter experts, key research personnel, and partners from ATSICCHOs and community-controlled organizations with expertise in Aboriginal and Torres Strait Islander health. Ethical approval from the relevant HREC will be outlined in research protocols (with inclusion/exclusion details) and obtained prior to data collection. Delphi participants will be invited to review the draft concepts and to indicate which ones should be prioritized or removed. This review process will be informed by Polit and Beck’s method [65], with each item ranked from 1 (Do not use) to 4 (Definitely keep). Scoring will be determined by summing the percentages of agreement between the panels; items that have a score greater than 79% will be included and those with scores less than 69% will be removed; those with in-between scores will be iteratively reviewed until a consensus is achieved (or for a maximum of 3 rounds). The accepted concepts will then be formatted into a document for further evaluation. Phase 5 is expected to be completed in June 2023.

### Phase 6: Expert Review II

Pragmatic feedback and discussion from Phase 5 will be used by the Collaboration to revise the set of principles that make up the framework using constant comparison to ensure that revisions are in line with Phases 1 to 4. A subsequent and final modified-Delphi exercise will be conducted with a broader, national panel of research experts and Aboriginal and Torres Strait Islander health professionals identified by the Collaboration. Ethical approval from the relevant HREC will be outlined in research protocols (with inclusion/exclusion details) and obtained prior to data collection. This final research phase will produce the foundational best practice framework for culturally safe eHealth interventions with Aboriginal and Torres Strait Islander people. Phase 6 is anticipated to be completed in September 2023.

### Phase 7: Dissemination

The foundational best practice framework will be disseminated in a scientific journal paper, together with an internal report to document the stages of research, evidence, feedback, authorship, and collaborations. Phase 7 is expected to be completed in December 2023.

## Results

Scoping work by members of the Collaboration with Aboriginal and Torres Strait Islander partners in 2019-2020 established interest and capacity for eHealth research projects including mHealth for hypertension management [87], Internet of Things to support independent living [88] and for housing suitability with climate change impacts [13]. Several of these feasibility projects commenced in 2020-2021 and qualitative data collection with research partners has commenced.

Conceptual discussion by the Collaboration for a best practice framework and the associated research program occurred in August 2020. A draft of the research program was produced in June 2021 with subsequent funding obtained in July 2021. The Collaboration approved the protocol draft in December 2021.

Results for several research phases of the best practice framework development are expected by January 2023, commencing with the systematic literature review and the scoping review. The projected completion times of subsequent phases are outlined in Figure 1, with the overall research program expected to be completed in December 2023.

## Discussion

### Key Anticipated Findings

The overall aim of this research program is to produce a best practice framework that will guide the co-design, implementation, and evaluation of culturally safe eHealth interventions with Aboriginal and Torres Strait Islander people. The development of the eHealth best practice framework will draw on evidence of best practices sourced from scientific and gray literature, health and community stakeholders, and key experts to reflect the values and priorities of Aboriginal and Torres Strait Islander people.

Maar et al [41] proposed a set of “wise practices” for cultural safety in eHealth with First Nations communities in 2019. The practices were developed from extensive qualitative data collected throughout a 5-year randomized clinical trial for managing hypertension with eHealth. Although the recommendations add significant value to the field, they are derived from works involving First Nations communities from Canada, and therefore cannot be directly applied to eHealth with Aboriginal and Torres Strait Islander people in the Australian setting.

Previous work involving Aboriginal and Torres Strait Islander people in the emerging digital health space have provided anecdotes regarding the important inclusions for such work. For example, favorable reports of user engagement, positive health outcomes, and the relevance of mHealth to support Aboriginal and Torres Strait Islander communities were attributed to the significance of co-design, embedded knowledge of Aboriginal and Torres Strait Islander people within projects, and consideration of local contexts rather than using a “one size fits all” approach [20]. However, more complete understanding and consensus are required on what is important to Aboriginal and Torres Strait Islander people when conducting eHealth interventions. Currently, no benchmark exists regarding the cultural safety within such studies and how eHealth can be effectively integrated within the existing models of care and leadership of ATSICCHOs. This integration is critical for maximum relevance and effectiveness in ensuring the well-being of Aboriginal and Torres Strait Islander people and for contributing to closing the gap in health disparities. This protocol has outlined a program of research designed by the Collaboration that seeks to meet this need.

Literature regarding the development of other guidelines within Aboriginal and Torres Strait Islander health settings offers insight into this research program. Although the approach of each research group to the development of guidelines varied given the differing health foci, there were parallels of significance [71,73-75]. Authentic consideration of culturally respectful research approaches with Aboriginal and Torres Strait Islander people was foundational. Research teams also highlighted the value of multiple sources for a quality evidence base, including literature, theory, and inputs from multiple stakeholder groups [71,73-75]. Reaching consensus from group-based activities was reported as a plausible challenge. Research teams worked through this by having further discussions with individual stakeholders and groups during subsequent rounds of consultation [73] or by recommending a discretionary approach in certain facets of applying the practice guidelines [71]. The challenge of capturing the diversity of all possible stakeholders considering remoteness, language, and literacy levels was acknowledged by authors [75].

### Strengths

This research program has several strengths. First, the protocol draws on a number of sources as an evidence base to shape the final framework. Beyond the thorough reviews of literature, best practice evidence will be gained from the expertise of Aboriginal and Torres Strait Islander leaders and health professionals, and insights provided by the Aboriginal and Torres Strait Islander people who are participants in eHealth research studies [56]. Second, the previous and ongoing eHealth research activities of the Collaboration across diverse settings provides governance for the research, along with an extensive network of research experts in the fields of eHealth and Aboriginal and Torres Strait Islander health. Both these strengths will ensure that the resulting best practice framework will not result from a single research team with a narrow frame of reference. Finally, the best practice phases are scheduled to occur over several years. This timeline will enhance the iterative process of data collection, theming, and stakeholder consultations with flexibility in the delivery of outputs.

### Limitations

The limitations of this research may involve either a dearth or an excess of evidence in the scoping and systematic reviews. This will be managed with guidance from the Collaboration as needed. The feasibility of conducting the consultation phases of this research may also be a challenge. Although the Collaboration has an excellent network of experts, a flexible approach will be necessary in light of the recent challenges related to travel and in-person sessions. For example, Delphi consensus sessions may need to be hybrid, including internet-based and in-person sessions.

Following dissemination, it will be important for the Collaboration to conduct ongoing promotion, evaluation, and adaptation processes of the best practice framework, as emphasized by Moher et al [63]. Application of the framework in future eHealth trials in partnership with ATSICCHOs and other Aboriginal and Torres Strait Islander stakeholders will generate evidence for its validity. Maintenance of a relevant and rigorous framework will be necessary as technology continues to evolve.

### Conclusions

This protocol has outlined the phases of a research program to prepare a best practice framework that will guide and inform the co-design, implementation, and evaluation of culturally safe eHealth interventions within existing models of health care for Aboriginal and Torres Strait Islander people. Data collected throughout the phases will be sourced from scientific literature, stakeholders, and the expertise of Aboriginal and Torres Strait Islander health sector, providing rigor and validity to the resulting framework.

It is timely that principles are generated to guide the overall eHealth research process, drawing on the excellence of Aboriginal and Torres Strait Islander primary health care models in real-world settings. The iterative and collaborative approach of this research program will also ensure that Aboriginal and Torres Strait Islander people determine the cultural safety and research relevance. Future research to validate the framework and monitor its relevance will be important.

## Appendix

Multimedia Appendix 1 Information and communication technologies in health care: definitions, health scenarios, and example products.

## Abbreviations

ATSICCHO: Aboriginal and Torres Strait Islander Community Controlled Health Organization
CONSORT: Consolidated Standards of Reporting Trials
HREC: Human Research Ethics Committee
mHealth: mobile health
PRISMA: Preferred Reporting Items for Systematic reviews and Meta-Analyses
PRISMA-ScR: Preferred Reporting Items for Systematic reviews and Meta-Analyses extension for Scoping Reviews
RQ: research question
SPIRIT: Standard Protocol Items: Recommendations for Interventional Trials
