# Correction: Culturally Safe eHealth Interventions With Aboriginal and Torres Strait Islander People: Protocol for a Best Practice Framework

**DOI:** 10.2196/43413

**Published:** 2022-10-18

**Authors:** Georgina R Chelberg, Kaley Butten, Ray Mahoney, eHRCATSIH Group

**Affiliations:** 1 Australian E-Health Research Centre Commonwealth Scientific and Industrial Research Organisation Herston Australia; 2 Centre for Online Health The University of Queensland Woolloongabba Australia; 3 School of Public Health The University of Queensland Herston Australia; 4 See Acknowledgments of the original article

In “Culturally Safe eHealth Interventions With Aboriginal and Torres Strait Islander People: Protocol for a Best Practice Framework” (JMIR Res Protoc 2022;11(6):e34904), the authors noted one error.

In the originally published article, Reference 23 incorrectly appeared as follows:

Mitchell-Box K, Braun KL. Fathers' thoughts on breastfeeding and implications for a theory-based intervention. J Obstet Gynecol Neonatal Nurs 2012 Nov;41(6):E41-E50. [doi: 10.1111/j.1552-6909.2012.01399.x] [Medline: 22861175]

In the corrected version, the correct Reference [23] has been updated as follows:

Couch D, Doherty Z, Panozzo L, Naren T, Burzacott J, Ward B, et al. The impact of telehealth on patient attendance and revenue within an Aboriginal Community Controlled Health Organisation during COVID-19. Australian Journal for General Practitioners 2021 Nov;50(11):851-855. [doi: 10.31128/AJGP-07-21-6060] [Medline: 34713288]

The correction will appear in the online version of the paper on the JMIR Publications website on October 14, 2022, together with the publication of this correction notice. Because this was made after submission to PubMed, PubMed Central, and other full-text repositories, the corrected article has also been resubmitted to those repositories.
