# Supplementary material for: Culturally Safe eHealth Interventions With Aboriginal and Torres Strait Islander People: Protocol for a Best Practice Framework
Source: JMIR Res Protoc. 2022 Jun 10;11(6):e34904. doi: 10.2196/34904 (PMC9233256; doi:10.2196/34904)
Supplement: Multimedia Appendix 1 [file resprot_v11i6e34904_app1.docx]

Multi-media Appendix 1: Information and communication technologies in health care: definitions, health scenarios and example products

| **Electronic health (eHealth);**  **digital health** | eHealth is an umbrella term that encompasses “…the cost-effective and secure use of information and communications technologies in support of health and health-related fields, including health-care services, health surveillance, health literature, and health education, knowledge and research…” [1, p.109] | | |
| --- | --- | --- | --- |
|  | **Definition** | **Health scenario** | **Example products** |
| **Mobile health (mHealth) including text messaging and mobile apps** | “…the use of mobile  devices – such as mobile phones, patient monitoring devices, personal digital assistants (PDAs)  and wireless devices – for medical and public health practice” [2, p.27]. | - Patients with cardiovascular disease can upload health data to a mobile app that also provides education and support via clinical access - Text messaging used for public health and health behaviour interventions e.g. smoking cessation - Mental health and wellbeing apps | ‘Cardihab’ [3]  ‘iBobbly’ [4]  ‘AIMHi Stay Strong’ [5]  ‘YourCall’ ^TM^ [6] |
| **Telehealth including videoconference** | “…involves an interaction between a health care provider and a patient when the two are  separated by distance. That interaction may take place in real time (synchronously), for example by telephone or by use of a video link. Or it may also take place asynchronously (store-and-forward),  when a query is submitted and an answer provided later; (secure) email is an example of this technique” [2, p.56]. | - General Practitioner appointment by telephone or videoconference - Fracture clinic by videoconference to discuss rehabilitation and review of Xray images - Videoconference appointment between consumer and psychologist | ‘Healthdirect’  ‘VideoCall’  ‘Coviu’  Specialised telehealth platforms |
| **Remote patient monitoring;**  **virtual care** | Collection of patient’s physiological data using manual or contactless upload for monitoring and assessment by clinician. [7] | - Diabetes management using a Bluetooth glucometer with data cloud storage enabling remote clinical review | REMODEL app [8]  MoTHER app [9]  Telstra Health |
| **Internet of Things (iOT);**  **Smart technology platforms** | “…refers to a kind of network to connect anything with the Internet- based on specified protocols over information sensing equipment to realize smart recognition, positioning, tracking, monitoring, and administration…” and has valuable applications in healthcare. | - Smart home technology with wireless sensors (motion, temperature, humidity, etc.) connected to internet and analytic platform to support independent living in the home | Smarter Safer Homes [11] |

References:

1. World Health Organization (WHO). (2005). Fifty-eighth world health assembly. Geneva: WHO.

2. World Health Organization (WHO). (2016). Global diffusion of eHealth: making universal health coverage achievable: report of the third global survey on eHealth. Geneva: WHO.

3. Varnfield M, Karunanithi M, Lee CK, Honeyman E, Arnold D, Ding H, et al. Smartphone-based home care model improved use of cardiac rehabilitation in postmyocardial infarction patients: results from a randomised controlled trial. Heart. 2014;100(22):1770-9.

4. Tighe J, Shand F, McKay K, McAlister TJ, Mackinnon A, Christensen H. Usage and Acceptability of the iBobbly App: Pilot Trial for Suicide Prevention in Aboriginal and Torres Strait Islander Youth. JMIR Ment Health. 2020;7(12):e14296.,

5. Dingwall KM, Sweet M, Cass A, Hughes JT, Kavanagh D, Howard K, et al. Effectiveness of Wellbeing Intervention for Chronic Kidney Disease (WICKD): results of a randomised controlled trial. BMC Nephrology. 2021;22(1):136.

6. Sharpe S, Kool B, Whittaker R, Lee AC, Reid P, Civil I, et al. Effect of a text message intervention to reduce hazardous drinking among injured patients discharged from a trauma ward: a randomized controlled trial. npj Digital Medicine. 2018;1(1).

7. Malasinghe LP, Ramzan N, Dahal K. Remote patient monitoring: a comprehensive study. Journal of Ambient Intelligence and Humanized Computing. 2019;10(1):57-76.

8. Menon A, Fatehi F, Bird D, Darssan D, Karunanithi M, Russell A, et al. Rethinking Models of Outpatient Specialist Care in Type 2 Diabetes Using eHealth: Study Protocol for a Pilot Randomised Controlled Trial. Int J Environ Res Public Health. 2019;16(6).

9. Varnfield M, Redd C, Stoney RM, Higgins L, Scolari N, Warwick R, et al. MheartTHer, an mHealth System to Support Women with Gestational Diabetes Mellitus: Feasibility and Acceptability Study. Diabetes Technol Ther. 2021;23(5):358-66.

10. Usak M, Kubiatko M, Shabbir MS, Viktorovna Dudnik O, Jermsittiparsert K, Rajabion L. Health care service delivery based on the Internet of things: A systematic and comprehensive study. International Journal of Communication Systems. 2020;33(2):e4179.p6

11. Zhang Q, Varnfield M, Higgins L, Smallbon V, Bomke J, O'Dwyer J, et al. Smarter Safer Homes Solution to Support Older People Living in Their Own Homes through Enhanced Care Models: Methodologies for a Stratified Randomized Controlled Trial. JMIR Preprints. 2021.
